# Genetic Variation in *NR1I2* and Palbociclib Safety and Prognosis in Advanced Breast Cancer: An Exploratory Multicenter Study

**DOI:** 10.3390/ijms27188310

**Published:** 2026-09-18

**Authors:** Cristina Arqueros, Marta Andrés, Sònia Servitja, Júlia Giner, Ariadna Tibau, Maria Borrell, Sergio Martinez-Recio, Agustí Barnadas, Juliana Salazar

**Affiliations:** 1 Department of Medical Oncology, Hospital de la Santa Creu i Sant Pau, 08041 Barcelona, Spain; mandresg@santpau.cat (M.A.); atibau@santpau.cat (A.T.);; 2 Spanish Group for Breast Cancer Research (GEICAM), 08041 Barcelona, Spain; sonia.servitja@geicam.org; 3 Department of Medicine, Faculty of Medicine, Universitat Autònoma de Barcelona, 08035 Barcelona, Spain; 4 Translational Medical Oncology Laboratory, Institut de Recerca Sant Pau (IR Sant Pau), 08041 Barcelona, Spain; 5 Medical Oncology, Hospital del Mar, 08003 Barcelona, Spain; 6 Hospital del Mar Research Institute, 08003 Barcelona, Spain; 7 Department of Medicine and Life Science, Universitat Pompeu Fabra, 08003 Barcelona, Spain; 8 Medical Oncology Department, Corporació Sanitària Parc Taulí, 08208 Sabadell, Spain; jginer@tauli.cat; 9 Program on Regulation, Therapeutics and Law (PORTAL), Division of Pharmacoepidemiology and Pharmacoeconomics, Department of Medicine, Brigham and Women’s Hospital, Harvard Medical School, Boston, MA 02120, USA; 10 Medical Oncology Department, Vall d’Hebron Hospital, 08035 Barcelona, Spain; 11 Breast Cancer Group, Vall d’Hebron Institute of Oncology (VHIO), 08035 Barcelona, Spain

**Keywords:** HR-positive, HER2-negative advanced breast cancer, palbociclib, *NR1I2*, pharmacogenetics, safety, survival

## Abstract

Palbociclib has shown survival benefits in hormone receptor (HR)-positive and human epidermal growth factor receptor 2 (HER2)-negative locally advanced or metastatic breast cancer. At present, however, no genetic predictors of palbociclib-related hematological toxicity have been validated for clinical use. The aim of this study was to evaluate whether genetic variants are associated with palbociclib toxicity and patient prognosis. Multicenter study of 113 patients with HR-positive, HER2-negative locally advanced or metastatic breast cancer treated with palbociclib plus endocrine therapy. Genotyping of DNA from blood samples was performed to determine 18 variants in seven target genes (*CYP3A4*, *SULT2A1*, *ABCB1*, *ABCG2*, *NR1I2*, *POR*, and *CDKN1B*). Association analyses were performed to assess the relationship between the genetic variants and treatment-related toxicity and survival outcomes. Sixty-seven patients (59.3%) developed grade 3/4 neutropenia, 62 (54.9%) experienced disease progression, and 43 (38.1%) died. Although none of the univariate associations remained significant after Bonferroni correction, the multivariate analysis, adjusted for clinical variables, revealed several nominally significant associations. The *NR1I2* gene variants rs6785049 (*p* = 0.01) and rs3732360 (*p* = 0.009) were associated with grade 3/4 neutropenia. The *NR1I2* rs6785049 variant was associated with palbociclib treatment interruption (*p* = 0.03) and with worse progression-free (*p* = 0.003) and overall survival (*p* = 0.02). The findings of this exploratory study suggest potential associations between the *NR1I2* rs6785049 variant and treatment toxicity and survival outcomes in patients with HR-positive, HER2-negative locally advanced or metastatic breast cancer treated with palbociclib. However, these hypothesis-generating findings need to be independently validated.

## 1. Introduction

Treatment advances have improved the management of breast cancer in recent years and targeted therapies play a key role in improving clinical outcomes. Cyclin-dependent kinase (CDK) 4 and 6 inhibitors are therapeutic agents used for both metastatic and early-stage breast cancer, particularly in patients with hormone receptor (HR)-positive and human epidermal growth factor receptor 2 (HER2)-negative disease [1]. Palbociclib is a CDK4/6 inhibitor administered in combination with aromatase inhibitors or selective estrogen receptor degraders (SERDs), with proven efficacy and a favorable safety profile [2,3]. Pivotal clinical trials have demonstrated that palbociclib improves progression-free survival (PFS) [2,3]. These clinical benefits are further supported by real-world data suggesting that palbociclib can also improve overall survival (OS) [4]. However, patient tolerance to palbociclib is heterogeneous and therefore challenging, with the most common adverse effects being hematological toxicities. The main dose-limiting toxicity is severe early neutropenia (≥grade 3), which has been observed in more than 60% of cases [5], thus requiring dose decreases and, in up to 10% of cases, treatment discontinuation [2].

Individual patient-related factors may explain some of the variability observed in palbociclib plasma concentrations [6], which can influence both treatment-related toxicity and survival outcomes. These patient-related factors may include germline genomic alterations that modulate the pharmacodynamics and pharmacokinetics of the drug. Palbociclib-mediated CDK4/6 inhibition prevents phosphorylation of the retinoblastoma protein (RB), leading to the suppression of E2F transcription factor activity and the subsequent downregulation of target genes involved in DNA replication and the cell cycle in cancer cells [7,8]. Aside from its role in controlling E2F activity, RB also regulates the cell cycle by modulating the cyclin-dependent kinase inhibitor p27^Kip1^, encoded by *CDKN1B* [7]. Clinical outcomes in patients treated with palbociclib are further influenced by the bioavailability of the drug, which is mediated by the transporters P-glycoprotein and breast cancer resistance protein (BCRP) [9], encoded by *ABCB1* and *ABCG2*, respectively, and by hepatic oxidation and sulfonation processes involving cytochrome P450 3A (CYP3A) and the enzyme sulfotransferase (SULT) 2A1 [8]. The expression of these transporters and enzymes is regulated by the transcription factor pregnane X receptor (PXR, *NR1I2*) [10]. Common genetic variation in *NR1I2* can modulate its transcriptional activity and has been associated with interindividual differences in treatment-related toxicity [11,12,13,14,15,16]. In particular, the intronic variant rs6785049 has been linked to variability in response to CYP3A4 substrate drugs [17]. In addition, CYP3A4 function depends on electron transfer from NADPH mediated by cytochrome P450 oxidoreductase (POR) [18].

Pharmacogenetic research on palbociclib remains relatively scarce [19,20,21,22,23], although significant associations have been reported, mainly for the *ABCB1* and *CYP3A4* variants with severe neutropenia [21]. However, all those studies present limitations, including the small sample size in one case series [20], the retrospective design of other studies [20,22,23], analyses that include different CDK4/6 inhibitors in combination [21], and the limited number of candidate genes evaluated [19,21,22]. In addition, none of those studies performed survival analyses. Therefore, association studies including additional genetic variants of genes involved in the mechanism of action and metabolic pathways of palbociclib are needed to identify potential predictors that could help to reduce severe neutropenia and improve survival outcomes in breast cancer patients.

In this context, the aim of this multicenter study was to investigate a comprehensive set of genetic variants involved in palbociclib activity and metabolism and their association with toxicity and survival in a well-defined cohort of homogeneously treated patients with breast cancer.

## 2. Results

### 2.1. Clinical Characteristics and Safety Analyses

Table S1 shows the treatment-related toxicities attributable to the combined treatment (palbociclib plus aromatase inhibitors or SERDs). Seventy patients required one or more dose reductions, distributed as follows: one dose reduction (n = 46, 40.7%), two dose reductions (n = 22, 19.5%), and three dose reductions followed by drug withdrawal (n = 2, 1.8%). Fifty-one patients (45%) required treatment interruption. Sixty-seven (59.3%) patients developed grade 3/4 neutropenia. The median (range) number of days from treatment initiation to the first episode of grade 3/4 neutropenia was 29 (7–1140) days. The median number of days from the first episode of grade 3/4 neutropenia to recovery (≤ grade 2 neutropenia) was 9 (0–64) days. Of the 67 patients with grade 3/4 neutropenia, 51 (76%) required a dose reduction of palbociclib (*p* < 0.001) and 37 (55%) required treatment interruption (*p* = 0.007).

Table S2 shows the full results of the univariate analysis for the clinical variables and their association with grade 3/4 neutropenia. The absolute neutrophil count (ANC) at cycle 1 on day 1 (C1D1) was significantly associated with grade 3/4 neutropenia. Patients with low pretreatment ANC levels were at higher risk of developing grade 3/4 neutropenia than patients with higher pretreatment levels (OR: 6.0, 95% CI: 2.22–16.10; *p* < 0.001). Numerical differences were found between the histological subtype and grade 3/4 neutropenia. Patients with invasive carcinoma of no special type had a higher risk of developing grade 3/4 neutropenia than patients with non-ductal histology (OR: 2.71, 95% CI: 0.96–7.68; *p* = 0.05). None of the clinical variables analyzed were significantly associated with thrombocytopenia or anemia.

### 2.2. Clinical Characteristics and Survival Analyses

Median follow-up was 61.1 months (95% CI: 57.6–65.6). During follow-up, 62 (54.9%) patients developed disease progression and 43 (38.1%) died. Median PFS was 45.1 months (95% CI: 30.0–60.1). The estimated 5-year PFS was 36% (95% CI: 24–48%). Median OS was not reached during the observation period of the study. The estimated OS rate at 5 years was 43% (95% CI: 29–57%).

Line of therapy, age at palbociclib initiation, and palbociclib dose interruption were significantly associated with PFS (Table S2). PFS was better in patients on first-line therapy than in those receiving other lines of therapy: 70.1 months (95% CI: 48.2–91.9) versus 20.9 months (95% CI: 14.2–27.7) (*p* = 0.003). Patients < age 75 had better PFS than those ≥ age 75: 70.1 months [95% CI: 38.6–NA] versus 29 months (95% CI: 18.1–56.1) (*p* = 0.03). Patients without treatment interruption had better PFS than those with treatment interruption: 70.1 months [95% CI: not available (NA)–NA] versus 29.0 months (95% CI: 7.68–50.35) (*p* = 0.04). The clinical variables significantly associated with OS were: line of therapy (*p* = 0.02), age at palbociclib initiation (*p* = 0.005), menopausal status (*p* = 0.03), and palbociclib dose interruption (*p* = 0.047) (Table S2). The estimated 5-year OS rate for each variable was as follows: first-line therapy: 50% (95% CI: 32–68%); other lines of therapy: 29% (95% CI: 5–53%); age < 75 years: 66% (95% CI: 55–78%); age ≥ 75 years: 22% (95% CI: 4–53%); pre-menopausal status: 56% (95% CI: 27–85%); post-menopausal status: 40% (95% CI: 24–56%); without treatment interruption: 52% (95% CI: 32–72%); and with treatment interruption: 34% (95% CI: 14–54%).

### 2.3. Genetic Variants and Neutropenia: Associations with NR1I2 rs6785049 and rs3732360

Table S3 summarizes the full results of the univariate analysis for the genetic variants and their association with grade 3/4 neutropenia. The variants rs6785049 (*p* = 0.002, recessive model for the G allele, AA–AG versus GG; intronic variant) and rs3732360 (*p* = 0.01, recessive model for the C allele, TT–TC versus CC; 3′-UTR variant) in the *NR1I2* gene were significantly associated with grade 3/4 neutropenia (Figure S1; see Section 4.4). Carriers of the rs6785049-A allele or rs3732360-T allele were more likely to have grade 3/4 neutropenia than non-carriers. Among patients with the rs6785049-A allele, 68.3% (n = 56) presented grade 3/4 neutropenia compared to 35.5% (n = 11) of patients with the GG genotype for this variant. For the rs3732360 variant, 64.2% (n = 61) of patients carrying the T allele presented grade 3/4 neutropenia versus 33.3% (n = 6) of patients with the CC genotype. The analysis of these SNPs, in which pretreatment ANC and histological subtype were included as covariates, showed that both SNPs were nominally statistically significant: *NR1I2* rs6785049 (OR: 3.7, 95% CI: 1.33–10.42; *p* = 0.01) and *NR1I2* rs3732360 (OR: 6.4, 95% CI: 1.59–25.8; *p* = 0.009). These associations remained significant in sensitivity analyses restricted to grade 3/4 neutropenia occurring within the first 56 days of treatment and in a time-to-first-event analysis, both in unadjusted and adjusted models (Table S4).

### 2.4. Genetic Variants and Other Toxicities: Thrombocytopenia and Anemia

In the univariate analyses, several variants showed nominally significant associations with thrombocytopenia: *NR1I2* rs3814055 (*p* = 0.049, recessive model for the T allele, CC–CT versus TT); *ABCG2* rs2231142 (*p* = 0.04); *CYP3A4* rs35599367 (*p* = 0.04); *ABCB1* rs2032582 (*p* = 0.02, dominant model for the C allele, CT–CA–TT–TA versus CC); and *ABCB1* rs1128503 (*p* = 0.02, dominant model for the G alle, GA–AA versus GG) (Table S3). None of these SNPs remained significant in the multivariate logistic regression analysis that included all of the SNPs.

We found a nominally significant association between the *ABCB1* rs1128503-G allele and a higher risk of anemia (*p* = 0.03, recessive model for the A allele, GG–GA versus AA) (Table S3).

### 2.5. Genetic Variants and Progression-Free Survival: Association with NR1I2 rs6785049

**Figure 1:**
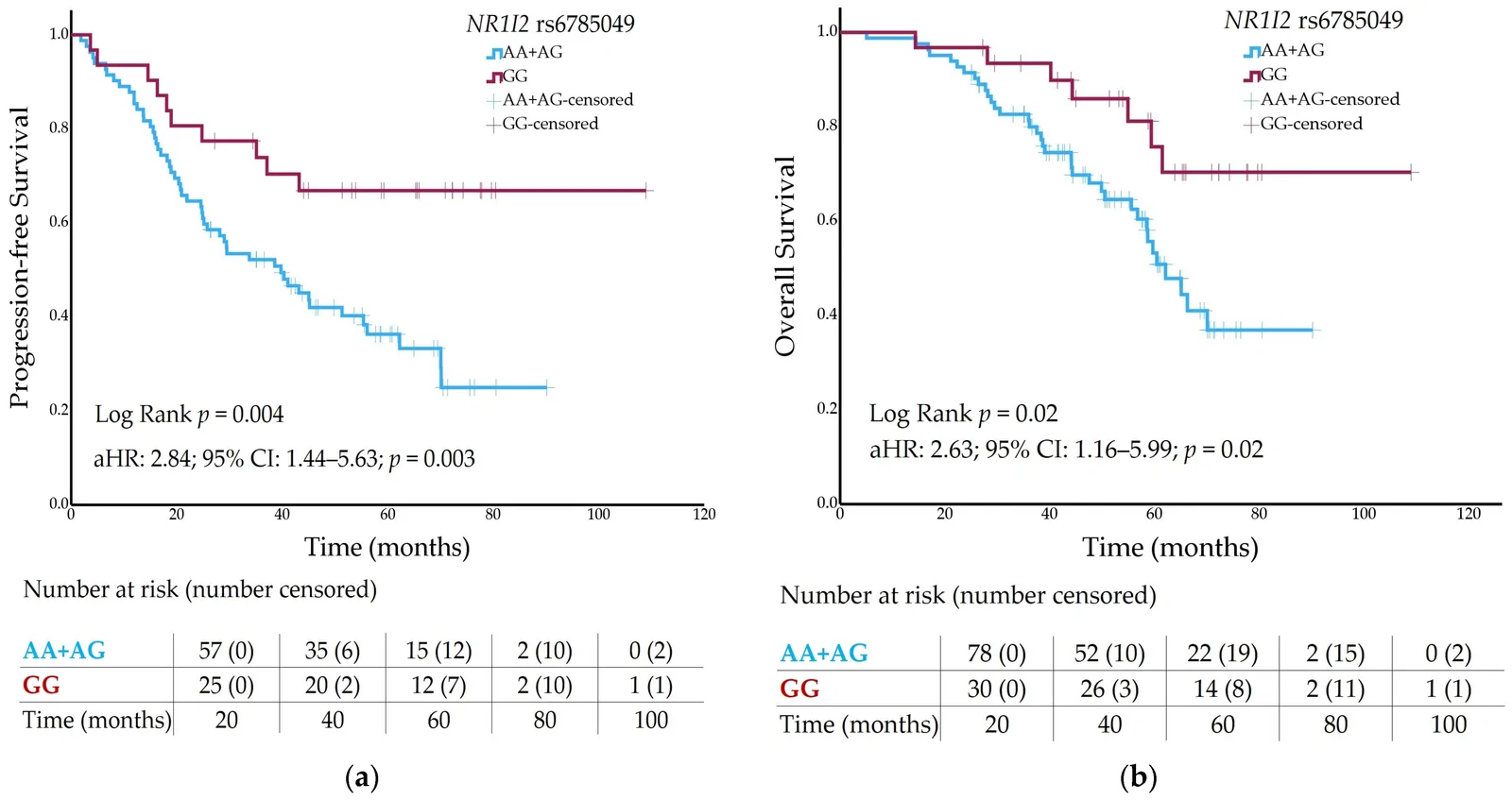
(**a**) Progression-free survival according to *NR1I2* rs6785049 genetic variant and (**b**) overall survival according to the *NR1I2* rs6785049 genetic variant in patients with hormone receptor (HR)-positive and human epidermal growth factor receptor 2 (HER2)-negative locally advanced or metastatic breast cancer. For each Kaplan–Meier curve, a table showing the number of patients at risk and the number censored at predefined time points is provided below the plot.

*NR1I2* rs6785049 and *POR* rs1057868 polymorphisms showed nominally significant associations with PFS on the Kaplan–Meier univariate analysis. The 5-year PFS was 67% (95% CI: 49–85%) for patients with the GG genotype versus 24% (95% CI: 10–38%) for carriers of the *NR1I2* rs6785049-A allele (*p* = 0.004, recessive model; Figure 1a). The 5-year PFS was 37% (95% CI: 25–49%) for carriers of the *POR* rs1057868-C allele versus 13% (95% CI: NA) for patients with the TT genotype (*p* = 0.03, recessive model). The full results of the univariate survival analyses for PFS are shown in Table S5. Additional Kaplan–Meier curves for *NR1I2* rs6785049 using a codominant genetic model are provided in Figure S2a.

In the multivariate Cox proportional hazards regression analysis for each SNP (adjusted for line of therapy, and age at palbociclib initiation as covariates), the *NR1I2* rs6785049 remained nominally significant [adjusted HR (aHR), 2.84; 95% CI: 1.44–5.63; *p* = 0.003]. However, the *POR* rs1057868 (aHR, 1.98; 95% CI: 0.86–4.56; *p* = 0.108) variant was no longer significant.

### 2.6. Genetic Variants and Overall Survival: Association with NR1I2 rs6785049

The *NR1I2* rs6785049 variant showed a nominally significant association with OS on the Kaplan–Meier univariate analysis. The 5-year OS was 66% (95% CI: 42–90%) for patients with the GG genotype compared to 34% (95% CI: 18–50%) for carriers of the A allele (*p* = 0.02, recessive model; Figure 1b). This association remained nominally statistically significant after adjusting for line of therapy, menopausal status, and age at palbociclib initiation as covariates (aHR, 2.63; 95% CI: 1.16–5.99; *p* = 0.02). Table S6 shows the full results of the univariate survival analyses for OS. Additional Kaplan–Meier curves for *NR1I2* rs6785049 using a codominant genetic model are provided in Figure S2b.

### 2.7. NR1I2 rs6785049 and Treatment Adjustment

To determine whether the *NR1I2* rs6785049 variant was also associated with treatment adjustment, we performed analyses that included palbociclib dose reduction and treatment interruption. In this analysis, the *NR1I2* rs6785049 variant was nominally associated with palbociclib treatment interruption (*p* = 0.03, recessive model). Patients carrying the A allele had more treatment interruptions than those with the GG genotype (52% versus 29%). Multivariate analyses that included line of therapy, menopausal status and age at palbociclib initiation as covariates showed a nominally significant association between *NR1I2* rs6785049 and treatment interruption (OR: 2.9, 95% CI: 1.15–7.46; *p* = 0.03) (Table 1). No significant associations were found between this genetic variant and palbociclib dose reduction (*p* = 0.52).

### 2.8. Statistical Correction for Multiple Hypothesis Testing

Although several associations reached nominal statistical significance, none remained significant after the Bonferroni correction (*p* < 0.0005) was applied to account for multiple comparisons.

### 2.9. Internal Validation: Associations of NR1I2 rs6785049

Internal bootstrap validation showed limited optimism across the adjusted logistic and Cox regression models assessing the associations of *NR1I2* rs6785049 with severe neutropenia, PFS, and OS. The optimism-corrected area under the receiver operating characteristic curve (AUROC) was 0.729 for severe neutropenia, while the optimism-corrected Harrell’s C-statistics were 0.631 for PFS and 0.675 for OS, compared with apparent estimates of 0.747, 0.653, and 0.697, respectively (Table S7). Bootstrap 95% CIs for the ORs and HRs are provided in Table S8.

## 3. Discussion

This study examined genetic variants and their association with severe neutropenia and survival outcomes in patients with locally advanced or metastatic breast cancer treated with palbociclib. The main findings suggest potential associations between the A allele for the NR1I2 rs6785049 variant and an increased risk of developing grade 3/4 neutropenia, palbociclib treatment interruption, and poorer PFS and OS.

CDK4/6 inhibitors administered in combination with endocrine therapy are the standard first-line treatment for metastatic and early-stage HR-positive and HER2-negative breast cancer. Combined treatment has been shown to yield significantly better PFS outcomes than endocrine therapy alone [24]. However, treatment response is variable and acquired resistance is common, which underscores the need to identify prognostic factors. Disease progression occurring within the first year of CDK4/6 inhibitor therapy has been associated with worse prognosis [25]. Several clinical studies, including the PARSIFAL-LONG trial [25] and the GEICAM-RegistEM study [26], have demonstrated the utility of testing for adjuvant hormone sensitivity to help select first-line treatment. Additionally, although palbociclib is generally well-tolerated, severe neutropenia is a common adverse effect that may compromise treatment [2,3,5]. To date, no genetic predictors have yet been identified and only limited pharmacogenetic research has been conducted in this area.

Iwata et al. [19] conducted a study involving patients from the pivotal PALOMA-2 and PALOMA-3 trials (palbociclib efficacy and safety trials). That study found associations between the *ABCB1* rs1128503 and *ERCC1* rs11615 variants and the risk of developing early grade 3/4 neutropenia; however, these variants had no impact on median PFS. In a small case series involving patients (n = 5) treated with palbociclib or ribociclib, Roncato et al. [20] measured the minimum plasma concentration (Cmin) for therapeutic drug monitoring. Those authors found that one of the patients who presented elevated plasma levels of the CDK4/6 inhibitor and severe neutropenia was a carrier of an *ABCB1* haplotype associated with reduced P-glycoprotein function, which may have contributed to the increase in systemic drug exposure. Conversely, they also found that a patient with subtherapeutic levels of the CDK4/6 inhibitor was a carrier of the CYP3A5 *1/*3 genotype, which may explain the enhanced metabolic activity that resulted in a lower plasma drug concentration. Peruzzi et al. [21] found that variants in the *ABCB1* gene and the CYP3A4*22 allele were associated with increased toxicity, including early severe neutropenia, early dose-limiting toxicity, and dose reductions in patients treated with the CDK4/6 inhibitors palbociclib, ribociclib, or abemaciclib.

Our study analyzed neutropenia during the follow-up period, in line with current clinical practice guidelines, in contrast to the previous studies [19,21] that evaluated grade 3/4 neutropenia at the first treatment cycle where only 20–30% of patients presented the toxicity. Severe neutropenia may occur earlier or later depending on baseline neutrophil counts, which have been shown to be a risk factor for the development of the toxicity both in the first cycle [19,21] and throughout more than one year of follow-up, as observed in our study. By evaluating neutropenia during the course of treatment, we were able to capture approximately 59% of severe neutropenia, providing a more comprehensive analysis of the overall extent of treatment-related neutropenia described in large randomized trials (~65%) [2,3].

The genetic variant *NR1I2* rs6785049 was associated with severe neutropenia and survival outcomes, although the mechanism through which this genetic variant may be involved in palbociclib response is currently unknown. CYP3A4 and SULT2A1 are the main enzymes responsible for palbociclib metabolism, while P-glycoprotein and BCRP proteins mediate drug transport [27]. All of these enzymes and proteins are encoded by genes regulated by the PXR transcription factor [10]. PXR expression has been reported to modulate the metabolism of chemotherapeutic agents such as irinotecan [28] and tamoxifen [29]. The *NR1I2* rs6785049 (A7635G) variant is located in intron 5 within the gene region that encodes the ligand-binding domain of the protein [17]. Although the variant is intronic, a two-fold increase in intestinal CYP3A induction after rifampicin exposure has been reported for the GG genotype compared to the AA genotype [17]. This suggests that the variant may have a regulatory role or may be in strong linkage disequilibrium with a causative variant.

To our knowledge, this is the first study to show that the *NR1I2* rs6785049 variant is associated with palbociclib toxicity. This finding is consistent with previous reports that have found an association between this variant and toxicity or pharmacokinetic parameters for other drugs metabolized via the same pathway [11,12,13]. In the study by Mbatchi et al. [12] involving patients with metastatic bladder cancer treated with temsirolimus, severe toxicities were more frequent in patients with higher area under the curve (AUC) values and in those with the *NR1I2* rs6785049 AA genotype. In a study involving renal transplant recipients, Miura et al. [13] found that plasma prednisolone concentrations, measured as AUC and maximum concentration (Cmax), were significantly lower in patients carrying the G allele than in those with the AA genotype for that variant. A study carried out in patients with chronic myeloid leukemia found that the plasma trough concentration (Ctrough) of bosutinib was lower in patients with the GG genotype than in carriers of the A allele for *NR1I2* rs6785049 [11]. Despite the significant associations reported in those studies, it is important to note that other authors have reported contradictory or negative findings [30,31,32].

The *NR1I2* rs6785049 variant was also associated with treatment interruption and poorer survival. In a study involving breast cancer patients treated with first-line palbociclib, Leenhardt et al. [33] found that the risk of developing grade 3/4 neutropenia was higher in patients with elevated palbociclib plasma Ctrough levels, suggesting a potential relationship between drug exposure and hematological toxicity. In our cohort, treatment interruption occurred in a significant percentage of patients who experienced grade 3/4 neutropenia. Notably, patients who experienced treatment interruption had significantly worse survival outcomes, consistent with previous studies [34,35]. However, given the observational design of this study, the associations observed between the variant and toxicity, treatment interruption, and survival outcomes should not be interpreted as causal, and the biological mechanisms underlying these associations remain unknown. Pharmacokinetic studies, including therapeutic drug monitoring in palbociclib-treated patients with known *NR1I2* rs6785049 genotypes, may provide additional evidence regarding these findings.

Future research could also explore the *NR1I2* rs6785049 variant in other biological and therapeutic settings to further investigate its potential relevance in advanced breast cancer treated with CDK4/6 inhibitors. The variant could be evaluated in endocrine-resistant luminal cancers treated with palbociclib in combination with PI3K pathway inhibitors [36], as activation of the PI3K/AKT/mTOR signaling axis is frequently associated with endocrine resistance [37,38]. Similarly, the variant could be assessed in patients with HR-positive, HER2-positive metastatic breast cancer receiving maintenance CDK4/6 inhibition combined with anti-HER2 therapy [39]. In these more complex therapeutic scenarios, where prolonged disease control and chronic treatment exposure are expected, optimizing dose intensity while minimizing severe toxicity is essential to maintain efficacy and preserve quality of life [24].

*NR1I2* rs3732360 was also associated with severe neutropenia. The rs3732360 variant is located in the 3′ UTR of *NR1I2* and has been reported to affect the binding sites of miR-500a-3p, miR-532-3p, and miR-374a-3p [14]. The base change may cause post-transcriptional dysregulation, resulting in altered PXR expression levels. Additionally, the T allele was associated with higher clearance of oral midazolam, a CYP3A4 substrate [40]. These findings suggest that the variant may be functional, although the biological mechanism underlying its association with palbociclib-related toxicity remains unknown.

This multicenter study of breast cancer patients homogeneously treated with palbociclib-based combination regimens and with an appropriate follow-up period provides novel evidence suggesting that genetic variability in *NR1I2* may be associated with treatment outcomes, including toxicity and survival. Additionally, it provides a rationale for prospective validation of the role of *NR1I2* rs6785049 in molecularly defined subgroups of patients with breast cancer receiving CDK4/6 inhibitor-based treatment. However, it has several limitations that should be considered. First, the findings of this pharmacogenetic study should be interpreted with caution, as none of the observed associations survived the Bonferroni correction for multiple comparisons. However, while the Bonferroni correction is an appropriate approach to account for multiple testing, it may be overly conservative in candidate gene studies. Additionally, although bootstrap-based internal validation was performed to assess model optimism, external validation in an independent cohort is needed to confirm these findings. Second, all patients were included retrospectively after treatment initiation, which may have introduced selection or survivor bias. Third, although the included genes were selected based on their relevance to palbociclib pharmacokinetics and pharmacodynamics and the genetic variants were supported by the literature, functional evidence is only available for a subset of these variants, and further functional studies are needed. Finally, the number of cases requiring palbociclib dose reduction due to grade 3/4 thrombocytopenia (n = 4) or grade 3/4 anemia (n = 3) was insufficient to perform association analyses, as expected given the incidence of these toxicities in this population. This limited our ability to determine the effect of the genetic variants on these severe toxicities. However, analyses based on the occurrence of thrombocytopenia (n = 61) and anemia (n = 88), regardless of grade, were performed instead.

In conclusion, this hypothesis-generating study investigated the association between genetic variants and both safety and survival outcomes in patients with HR-positive, HER2-negative locally advanced and metastatic breast cancer, with *NR1I2* rs6785049 emerging as a potential pharmacogenetic biomarker for palbociclib that requires validation in independent cohorts.

## 4. Materials and Methods

### 4.1. Study Design and Population

This was a retrospective, observational, multicenter study conducted at three hospitals in Spain: Hospital de la Santa Creu i Sant Pau (HSCSP; Barcelona), Hospital del Mar (Barcelona), and Hospital Parc Taulí (Sabadell, Barcelona). A total of 113 consecutive female patients diagnosed with HR-positive, HER2-negative locally advanced or metastatic breast cancer treated with palbociclib as first-, second-, or third-line therapy were enrolled between 2018 and 2022. Table 2 shows the clinical variables recorded: age at diagnosis, histology, menopausal status, progesterone receptor status, metastatic breast cancer, visceral metastasis, endocrine therapy, line of therapy, baseline ANC, palbociclib dose reduction, and dose interruption. Data cut-off was 30 March 2025.

### 4.2. Treatment Protocol

The treatment dosing schedule for palbociclib in all patients included in the study was 125 mg orally once daily for 21 consecutive days followed by 7 days off in combination with aromatase inhibitors (letrozole/exemestane/anastrozole) or fulvestrant at 500 mg on days 1, 15, 29, and once monthly thereafter. Exclusion criteria were male sex; triple-negative or HER2-positive breast cancer; concomitant use of CYP3A inhibitors or inducers; prophylactic granulocyte colony-stimulating factor (G-CSF) use; and life-threatening visceral metastatic disease, defined as extensive hepatic involvement, brain or leptomeningeal involvement (past or present), symptomatic pulmonary lymphangitic spread, or known bone marrow infiltration due to breast cancer. All patients had adequate hepatic and renal function at baseline, in accordance with the eligibility criteria for palbociclib treatment.

### 4.3. Outcome and Data Evaluation

The main outcomes were severe neutropenia (grade 3/4), PFS, and OS.

Toxicity data were collected from clinical records. The following toxicities were registered: neutropenia, leukopenia, anemia, thrombocytopenia, and febrile neutropenia. Neutropenia, anemia and thrombocytopenia were graded according to the Common Terminology Criteria for Adverse Events (CTCAE, version 5) [41]. The highest grade of neutropenia during the course of treatment was used for the analyses and dichotomized into grades 0/1–2 and grades 3–4. A cutoff for ANC values of 3.6 × 10^3^/mm^3^ at C1D1 was established based on previous studies [21,42]. Anemia and thrombocytopenia were defined as present (grades 1–4) or absent (grade 0) and coded as yes/no, as only three cases of grades 3–4 anemia and four of grade 3 thrombocytopenia were observed (see Table S1). Dose reduction (yes/no) was defined as a reduction of the palbociclib dose from 125 mg to 100 or 75 mg.

Disease progression was assessed radiologically according to RECIST v1.1 criteria. Computed tomography (CT) and bone scintigraphy were performed for radiological assessment in all patients.

PFS was defined as the time from the start of combined treatment (palbociclib plus fulvestrant or aromatase inhibitors) to first evidence of disease progression or death by any cause, whichever occurred first. OS was defined as the time from initiation of combined treatment to death from any cause. Patients without event were censored at the date of last disease assessment.

### 4.4. Genetic Variant Selection and Genotyping

Genes encoding for proteins involved in the metabolism (*CYP3A4* and *SULT2A1*) and transport (*ABCB1* and *ABCG2*) of palbociclib, and for regulators of these proteins (*NR1I2* and *POR*) or the cell cycle (*CDKN1B*) were selected. The genetic variants that could potentially affect the protein activities of the candidate genes according to the published literature (see Table 3) and/or according to the RegulomeDB [43] and HaploReg [44] bioinformatics databases were determined.

Blood samples were collected at study enrollment. Genomic DNA was extracted from peripheral whole-blood samples. Genotyping was performed using real-time polymerase chain reaction (PCR) on a 7900 HT Real-Time PCR System (Applied Biosystems; Foster City, CA, USA). Single-nucleotide polymorphisms (SNPs) were analyzed using TaqMan^®^ SNP genotyping assays ((Applied Biosystems; Foster City, CA, USA; Table S9). *SULT2A1* copy number variations (CNVs) were analyzed in triplicate using TaqMan^®^ copy number assay and RNase P (Applied Biosystems, Foster City, CA, USA). Relative quantification of CNVs was performed with CopyCaller™ software, v.2.1 (Applied Biosystems; Foster City, CA, USA). All procedures were performed according to the manufacturers’ instructions.

The allele frequencies observed in this study were comparable to the rates reported in the European population [52]. All samples and SNPs were successfully genotyped. The genotypic distribution for each SNP was in Hardy–Weinberg equilibrium, with the exception of *CYP3A4* rs2740574 (Table S9). Given the low frequency of this variant (3%) and the small number of homozygous individuals (one sample was homozygous for the *CYP3A4* variant, whereas the expected number was zero), the SNP was conservatively excluded from the analyses.

### 4.5. Statistical Analysis

The estimated statistical power of the study to detect small-to-moderate effect sizes (w = 0.2–0.3; α = 0.05) ranged from 57% to 89% (G*power, v. 3.1.9.7, Düsseldorf, Germany) [53]. For the triallelic *ABCB1* rs2032582 variant, genotypes were categorized as GG (wild-type), GT or GA (heterozygous), and TT, TA or AA (carriers of two low-frequency alleles). This grouping was used to enable cross-tabulation analyses. For *SULT2A1*, individuals with 0 and 1 copy were grouped into a single category because only two individuals had 0 copies. All SNPs were initially analyzed under a codominant genetic model. For SNPs showing suggestive evidence of association under the codominant model (*p* ≤ 0.1), alternative dominant or recessive inheritance models were explored to characterize potential genotype–phenotype patterns. Differences in toxicities were calculated using the Wald chi-square test or Fisher’s exact test depending on the sample size. Clinically relevant covariates [2,3] were assessed in univariate analyses, with statistically significant variables included in the multivariable models (Table 2). Treatment interruption was not included in the genotype–survival models because it is a post-baseline variable and may represent a mediator of the genotype–survival association. Logistic regression analyses for grade 3/4 neutropenia were performed using the histological subtype (invasive carcinoma of no special type versus non-ductal) and ANC at C1D1 of treatment (<3.6 × 10^3^/mm^3^ versus ≥3.6 × 10^3^/mm^3^) as covariates, as these variables were significant in the univariate analyses. The results are expressed as odds ratios (ORs) with 95% confidence intervals (CIs). To assess potential exposure-time bias arising from ascertainment of neutropenia over the entire treatment period, two sensitivity analyses were performed under the recessive genetic model: early grade 3/4 neutropenia landmark analysis (defined as grade 3/4 neutropenia occurring within the first 56 days of treatment -up to cycle 3, day 1) was analyzed by logistic regression; and time to first grade 3/4 neutropenia episode (patients who did not develop grade 3/4 neutropenia were censored at the last treatment cycle received) was analyzed using Cox proportional hazards regression and log-rank test; both outcomes were analyzed unadjusted and adjusted for baseline ANC and histological subtype. The Kaplan–Meier method was used for the survival analyses. Between-group survival differences were compared using the log-rank test. Cox proportional hazards regression models were used for the multivariate analysis. Covariates were selected based on their significance in the univariate analyses. For OS, line of therapy (“other” versus first-line treatment), menopausal status (post-menopausal versus pre-menopausal), and age at palbociclib initiation (≥75 versus <75 years) were included. For PFS, line of therapy (“other” versus first-line treatment) and age at palbociclib initiation (≥75 versus <75 years) were included. The reverse Kaplan–Meier was used to calculate median follow-up. The results are expressed as hazard ratios (HR) and 95% CIs. The proportional hazards assumption was assessed using Schoenfeld residuals; no evidence of a violation of the proportional hazards assumption was observed (global test, *p* = 0.78). Associations with *p* < 0.05 were considered nominally significant. To account for multiple testing, a Bonferroni-adjusted significance threshold of *p* < 0.0005 was applied based on 94 independent univariate association tests performed, including survival analyses. All *p*-values reported in the manuscript are unadjusted, whereas the corresponding Bonferroni-adjusted *p*-values are provided in the Supplementary Tables S3, S5, and S6. Statistical analyses were performed with the IBM-SPSS Statistical software, v. 29.0. Internal validation was performed using bootstrap resampling with 2000 resamples. The prespecified multivariable logistic and Cox regression models were refitted in each bootstrap sample. Bootstrap 95% CIs were calculated for the ORs and HRs. Model discrimination was assessed using the AUROC for the logistic regression model and Harrell’s C-statistic for the Cox models. Optimism-corrected performance estimates were obtained by subtracting the mean optimism estimated from the bootstrap resamples from the apparent model performance.

## Figures and Tables

**Table 1:** Multivariate significant associations between the *NR1I2* rs6785049 genetic variant and treatment toxicity and survival outcomes (recessive model) in patients with hormone receptor (HR)-positive and human epidermal growth factor receptor 2 (HER2)-negative locally advanced or metastatic breast cancer.

	**Grade 3/4 Neutropenia (n = 103)**		**Treatment Interruption (n = 111)**	
***NR1I2* rs6785049**	**n (%)**	**OR (95% CI)**	**n (%)**	**OR (95% CI)**
AA	21 (63.6)		16 (48.5)	
AG	35 (71.4)		26 (54.2)	
GG	11 (35.5)		9 (29)	
GG	11 (35.5)	Reference (1)	9 (29)	Reference (1)
AA–AG	56 (68.3)	3.7 * (1.33–10.42)	42 (51.9)	2.9 * (1.15–7.46)
	**Progression-Free Survival (n = 111)**		**Overall Survival (n = 112)**	
***NR1I2* rs6785049**	**Probability** **(95% CI) at 5-Years**	**Adjusted HR** **(95% CI)**	**Probability** **(95% CI) at 5-Years**	**Adjusted HR** **(95% CI)**
AA	0.28 (0.03–0.53)		0.46 (0.19–0.73)	
AG	0.21 (0.05–0.37)		0.28 (0.08–0.48)	
GG	0.67 (0.49–0.85)		0.66 (0.42–0.90)	
GG	0.67 (0.49–0.85)	Reference (1)	0.66 (0.42–0.90)	Reference (1)
AA–AG	0.24 (0.10–0.38)	2.84 ** (1.44–5.63)	0.34 (0.18–0.50)	2.63 * (1.16–5.99)

HR, hazard ratio; OR: odds ratio; CI: confidence intervals; * *p* < 0.05; ** *p* < 0.01.

**Table 2:** Baseline characteristics and treatment details of patients with hormone receptor (HR)-positive and human epidermal growth factor receptor 2 (HER2)-negative locally advanced or metastatic breast cancer (N = 113).

Characteristic		n	%
Age at diagnosis, median (range) years		61 (38–86)	
Age at diagnosis	<75 years	92	81.4
	≥75 years	19	16.8
	Unknown	2	1.8
Age at palbociclib initiation, median (range) years		65 (38–87)	
Age at palbociclib initiation	<75 years	90	79.6
	≥75 years	23	20.4
Histology	Ductal	90	79.6
	Non-ductal	18	15.9
	Unknown	5	4.4
Menopausal status	Pre-menopausal	26	23.0
	Post-menopausal	86	76.1
	Unknown	1	0.9
Progesterone receptors	Yes	100	88.5
	No	13	11.5
Metastatic breast cancer	De novo	33	29.2
	Relapsed	80	70.8
Visceral metastases	Yes	62	54.9
	No	51	45.1
Endocrine therapy	AI	71	62.8
	SERD	42	37.2
Line of therapy	First	78	69.0
	Second	27	23.9
	Third	8	7.1
Baseline ANC	<3.6 × 10^3^/mm^3^	38	33.6
	≥3.6 × 10^3^/mm^3^	70	61.9
	Unknown	5	4.4
Palbociclib dose reduction	Yes	70	61.9
	No	43	38.1
Palbociclib dose interruption	Yes	51	45.0
	No	61	54.1
	Unknown	1	0.9
Center	HSCSP	61	54.0
	Hospital del Mar	32	28.3
	Hospital Parc Taulí	20	17.7
Race	Caucasian	107	94.7
	Non-Caucasian	6	5.3

AI, aromatase inhibitors; SERD, selective estrogen receptor degrader; ANC, absolute neutrophil count; and HSCSP, Hospital de la Santa Creu i Sant Pau.

**Table 3:** Characteristics of the polymorphisms studied.

Gene	rsID; PGx Allele	Location or Effect	References
*CYP3A4*	rs2740574; CYP3A4*1B	2KB Upstream variant	[45]
	rs35599367; CYP3A4*22	Intron variant	
*SULT2A1*	rs182420	Downstream variant	[23,46]
	rs2637125	Upstream variant	
	CNV		
*ABCG2*	rs2231142	p.Gln141Lys	[23]
*ABCB1*	rs1045642	p.Ile1145=	[21,47]
	rs2032582	p.Ser893Thr; p.Ser893Ala	
	rs1128503	p.Gly412=	
*NR1I2*	rs3814055	2KB Upstream variant	[15,16]
	rs2472677	Intron variant	
	rs6785049	Intron variant	
	rs3814058	3′-UTR variant	
	rs3732360	3′-UTR variant	
	rs1054190	3′-UTR variant	
	rs7643645	Intron variant	
*POR*	rs1057868; POR*28	p.Ala503Val	[18,48,49,50]
*CDKN1B*	rs2066827	p.Val109Gly	[51]

rsID, reference single-nucleotide polymorphism identifier; PGx, pharmacogenetic; and CNV, copy number variation.

## Data Availability

The data presented in this study are available on request from the corresponding author due to ethical committee regulations.

## Supplementary Materials

The following supporting information can be downloaded at: https://www.mdpi.com/article/10.3390/ijms27188310/s1.

## Abbreviations

The following abbreviations are used in this manuscript:

CYP3A4	Cytochrome P450 family 3 subfamily A member 4
SULT2A1	Sulfotransferase family 2A member 1
ABCG2	ATP binding cassette subfamily G member 2
ABCB1	ATP binding cassette subfamily B member 1
*NR1I2* (PXR)	Nuclear receptor subfamily 1 group I member 2
POR	Cytochrome P450 oxidoreductase
CDKN1B	Cyclin-dependent kinase inhibitor 1B
