# Supplementary material for: Genetic Variation in NR1I2 and Palbociclib Safety and Prognosis in Advanced Breast Cancer: An Exploratory Multicenter Study
Source: Int J Mol Sci. 2026 Sep 18;27(18):8310. doi: 10.3390/ijms27188310 (PMC13607729; doi:10.3390/ijms27188310)
Supplement: Supplementary file 1 [file ijms-27-08310-s001.zip › ijms-4529137-supplementary.pdf]

Supplementary Material

Genetic Variation in *NR1I2* and Palbociclib Safety and Prognosis in Advanced Breast Cancer: An Exploratory Multicenter Study

Authors:

Cristina Arqueros [1,2,3,4,*,†], Marta Andrés [1,2,3,4,†], Sònia Servitja [2,5,6], Júlia Giner [7], Ariadna Tibau [1,4,8], Maria Borrell [9,10], Sergio Martinez-Recio [1,4], Agustí Barnadas [1,4] and Juliana Salazar [4,*]

Table S1. Palbociclib-related adverse events in patients with hormone receptor (HR)-positive and human epidermal growth factor receptor 2 (HER2)-negative locally advanced or metastatic breast cancer.

Toxicities	No-toxicity	Toxicity			
	n (%)	Grades	n (%)	Grades	n (%)
Neutropenia	5 (4.4)	G1	15 (13.3)	G3	63 (55.8)
		G2	26 (23.0)	G4	4 (3.5)
Thrombocytopenia	52 (46.0)	G1	55 (48.7)	G3	4 (3.5)
		G2	2 (1.8)	G4	0 (0)
Anemia	25 (22.1)	G1	65 (57.5)	G3	2 (1.8)
		G2	20 (17.7)	G4	1 (0.9)
Febrile neutropenia*	110 (97.3)				

G, grade. *Febrile neutropenia was reported in three patients.

Table S2. Univariate associations between clinical covariates and severe neutropenia, progression-free survival and overall survival in patients with hormone receptor (HR)-positive and human epidermal growth factor receptor 2 (HER2)-negative locally advanced or metastatic breast cancer.

		**Grade 3/4 neutropenia**			**Progression-free survival**			**Overall survival**		
**Variable**	**n**	**n (%)**	**OR (95%CI)**	***p*-value**	**Probability (95% CI) at 5-years**	**HR (95% CI)**	***p*-value (log-rank)**	**Probability (95% CI) at 5-years**	**HR (95% CI)**	***p*-value (log-rank)**
Age at diagnosis				0.51			**0.01**			**<0.001**
<75 years	92	56 (60.9)	1		0.42 (0.28 - 0.56)	1		0.5 (0.34 - 0.66)	1	
≥75 years	19	10 (52.6)	0.71 (0.26-1.93)		0.07 (NA - 0.25)	2.09 (1.16 - 3.74)		0.14 (NA - 0.36)	3.02 (1.56 - 5.84)	
Age at start				0.44			**0.03**			**0.005**
<75 years	90	55 (61.1)	1		0.5 (0.4-0.61	1		0.66 (0.55-0.78)	1	
≥75 years	23	12 (52.2)	0.69 (0.28-1.74)		0.22 (0.02-0.42)	1.87 (1.06-3.28)		0.29 (0.04-0.53)	2.51 (1.30-4.84)	
Histology				**0.05**			0.46			0.74
Ductal	90	57 (63.3)	2.71 (0.96-7.68)		0.34 (0.20 - 0.48)	1.30 (0.64 -2.65)		0.4 (0.24 - 0.56)	1.16 (0.49 - 2.75)	
Non-ductal	18	7 (38.9)	1		0.44 (0.19 - 0.69)	1		0.59 (0.32 - 0.86)	1	
Menopausal status							0.53			**0.03**
pre-menopausal	26	18 (69.2)	1	0.22	0.37 (0.12 - 0.62)	1		0.56 (0.27 - 0.85)	1	
post-menopausal	86	48 (55.8)	0.56 (0.22-1.43)		0.37 (0.23 - 0.51)	1.22 (0.66 - 2.26)		0.4 (0.24 - 0.56)	2.49 (1.05 - 5.92)	
Progesterone receptors				0.86			0.65			0.86
Yes	100	59 (59)	1		0.37 (0.25 - 0.49)	1		0.44 (0.30 -0.58)	1	
No	13	8 (61.5)	1.11 (0.34-3.64)		0.17 (NA - 0.56)	1.21 (0.52 - 2.83)		0.32 (NA - 0.79)	1.1 (0.39 - 3.09)	
Metastatic breast cancer				0.86			0.92			0.97
de novo	33	20 (60.6)	1		0.36 (0.14 - 0.58)	1		0.42 (0.17 - 0.67)	1	
relapsed	80	47 (58.8)	0.93 (0.40-2.12)		0.36 (0.22 - 0.50)	1.03 (0.59 -1.78)		0.44 (0.28 - 0.60)	1.01 (0.53 - 1.94)	

Visceral metastases				0.93			0.27			0.64
Yes	62	37 (59.7)	1.04 (0.49-2.2)		0.3 (0.12 - 0.48)	1.33 (0.8 -2.21)		0.38 (0.18 - 0.58)	1.15 (0.63 -2.11)	
No	51	30 (58.8)	1		0.41 (0.23 - 0.59)	1		0.47 (0.27 - 0.67)	1	
Endocrine therapy				0.72			0.17			0.61
AI	71	43 (60.6)	1		0.38 (0.22 - 0.54)	1		0.48 (0.30 - 0.66)	1	
SERD	42	24 (57.1)	0.87 (0.40-1.88)		0.33 (0.17 - 0.49)	1.42 (0.85 -2.36)		0.35 (0.11 - 0.59)	1.18 (0.63 - 2.19)	
Line of therapy				0.35			**0.003**			**0.02**
First	78	44 (56.4)	1		0.41 (0.25 - 0.57)	1		0.5 (0.32 - 0.68)	1	
Others	35	23 (65.7)	1.48 (0.65-3.39)		0.25 (0.09 -0.41)	2.16 (1.29 - 3.61)		0.29 (0.05 - 0.53)	2.03 (1.09 - 3.76)	
Baseline ANC				**<0.001**						
$<3.6x10^3/mm^3$	38	32 (84.2)	6.0 (2.22-16.10)							
$\geq3.6x10^3/mm^3$	70	33 (47.1)	1							
Palbociclib dose reductions							0.99			0.88
Yes	70				0.35 (0.19 - 0.51)	1.00 (0.6 - 1.68)		0.43 (0.25 - 0.61)	1.05 (0.57 - 1.94)	
No	43				0.36 (0.16 - 0.56)	1		0.44 (0.22 - 0.66)	1	
Palbociclib dose interruptions							**0.04**			**0.047**
Yes	51				0.27 (0.11 -0.43)	1.71 (1.03 - 2.83)		0.34 (0.14 - 0.54)	1.84 (0.99 - 3.40)	
No	61				0.45 (0.27 - 0.63)	1		0.52 (0.32 - 0.72)	1	

AI, Aromatase inhibitors; SERD, Selective estrogen receptor degraders; ANC, absolute neutrophil count; Statistically significant *p*-values are marked in bold.

Table S3. Univariate associations between genetic variants and palbociclib-related adverse events in patients with hormone receptor (HR)-positive and human epidermal growth factor receptor 2 (HER2)-negative locally advanced or metastatic breast cancer.

		Grade 3/4 neutropenia				Thrombocytopenia				Anemia			
**SNP**	**n**	**n (YES)**	**%**	***p*-value**	**Bonferroni-adjusted *p*-value**	**n (YES)**	**%**	***p*-value**	**Bonferroni-adjusted *p*-value**	**n (YES)**	**%**	***p*-value**	**Bonferroni-adjusted *p*-value**
NR1I2 rs3814055				0.39				0.09				0.59	
CC	36	18	50			16	44.4			29	80.6		
CT	63	40	63.5			34	54.0			47	74.6		
TT	14	9	64.3			11	78.6			12	85.7		
CC – CT[b]						50	50.5	**0.049**	1.00				
NR1I2 rs2472677				0.77				0.66				0.95	
TT	39	23	59			19	48.7			31	79.5		
TC	56	32	57.1			31	55.4			43	76.8		
CC	18	12	66.7			11	61.1			14	77.8		
NR1I2 rs6785049				**0.005**	0.47			0.11				0.16	
AA	33	21	63.6			21	63.6			25	75.8		
AG	49	35	71.4			21	42.9			42	85.7		
GG	31	11	35.5			19	61.3			21	67.7		
AA - AG[b]	82	56	68.3	**0.002**	0.19								
NR1I2 rs3814058				0.42				0.10				0.81	
TT	58	35	60.3			33	56.9			46	79.3		
TC	45	28	62.2			20	44.4			35	77.8		
CC	10	4	40			8	80			7	70		
NR1I2 rs3732360				**0.04**	1.0			0.15				0.12	
TT	49	30	61.2			29	59.2			38	77.6		
TC	46	31	67.4			20	43.5			39	84.8		

CC	18	6	33.3			12	66.7			11	61.1	
TT - TC[b]	95	61	64.2	**0.01**	0.94							
NR1I2 rs1054190				0.08				0.26				0.40
CC	88	56	63.6			50	56.8			67	76.1	
CT - TT	25	11	44			11	44			21	84	
NR1I2 rs7643645				0.86				0.65				0.81
AA	48	29	60.4			26	54.2			36	75	
AG	51	29	56.9			29	56.9			41	80.4	
GG	14	9	64.3			6	42.9			11	78.6	
SULT2A1 rs182420				0.46				0.98				0.95
TT	63	37	58.7			34	54.0			49	77.8	
TC	44	25	56.8			24	54.5			34	77.3	
CC	6	5	83.3			3	50			5	83.3	
SULT2A1 rs2637125				0.31				0.76				0.15
GG	82	51	62.2			45	54.9			61	74.4	
GA - AA	29	16	51.6			16	51.6			27	87.1	
SULT2A1 CNV				0.51				0.45				0.52
2 copies	80	49	61.3			45	56.3			61	76.3	
0-1 copies	33	18	54.5			16	48.5			27	81.8	
ABCG2 rs2231142				0.45				**0.04**	1.00			0.19
GG	99	60	60.6			57	57.6			79	79.8	
GT	14	7	50			4	28.6			9	64.3	
CDKN1B rs2066827				0.98				0.09				0.87
TT	64	38	59.4			38	59.4			51	79.7	
TG	41	24	58.5			17	41.5			31	75.6	
GG	8	5	62.5			6	75.0			6	75	
CYP3A4 rs35599367				0.24				**0.04**	1.00			0.39
GG	102	59	57.8			52	51.0			78	76.5	

GA	9	7	77.8		8	88.9			8	88.9		
ABCB1 rs1045642				0.59			0.08				0.64	
GG	30	18	60.0		20	66.7			25	83.3		
GA	61	34	55.7		27	44.3			47	77		
AA	22	15	68.2		14	63.6			16	72.7		
ABCB1 rs2032582				0.83			**0.05**	1.00			0.23	
CC	37	23	62.2		26	70.3			31	83.8		
CT - CA	60	34	56.7		27	45.0			47	78.3		
TT - TA	16	10	62.5		8	50			10	62.5		
CT - CA -TT - TA[a]					35	46.1	**0.02**	1.00				
ABCB1 rs1128503				0.95			0.08				0.1	
GG	36	22	61.1		25	69.4			30	83.3		
GA	57	33	57.9		27	47.4			46	80.7		
AA	20	12	60		9	45			12	60		
GA – AA[a]	77				36	46.8	**0.02**	1.00				
GG - GA[b]	93								76	81.7	**0.03**	1.00
POR rs1057868				0.15			0.2				0.45	
CC	58	38	65.5		34	58.6			47	81		
CT	47	23	48.9		25	53.2			34	72.3		
TT	8	6	75		2	25			7	87.5		

SNP, single-nucleotide polymorphism; [a]dominant genetic model; [b]recessive genetic model. Nominally statistically significant *p*-values are marked in bold.

Table S4. Sensitivity analyses for the association between *NR1I2* rs6785049 and rs3732360 variants and grade 3/4 neutropenia in patients with hormone receptor (HR)-positive and human epidermal growth factor receptor 2 (HER2)-negative locally advanced or metastatic breast cancer.

**Genotype (recessive model)**	**n**	**Early grade 3/4 neutropenia (≤56 days, up to C3D1)**			**Time to first grade 3/4 neutropenia**	
		**n (%)**	**Unadjusted OR (95% CI); *p***	**Adjusted OR (95% CI); *p* [a]**	**Unadjusted HR (95% CI); *p* [b]**	**Adjusted HR (95% CI); *p* [a]**
***NR1I2* rs6785049**						
**GG (ref)**	31	7 (22.6)	1	1	1	1
**AA–AG**	82	46 (56.1)	4.38 (1.70–11.31); 0.002	4.36 (1.60–11.84); 0.004	2.83 (1.47–5.48); 0.001	2.66 (1.37–5.19); 0.004
***NR1I2* rs3732360**						
**CC (ref)**	18	4 (22.2)	1	1	1	1
**TT–TC**	95	49 (51.6)	3.73 (1.14–12.16); 0.029	4.69 (1.31–16.88); 0.018	2.95 (1.26–6.90); 0.009	3.49 (1.47–8.28); 0.005

OR, odds ratio; HR, hazard ratio; CI, confidence interval; ANC, absolute neutrophil count; C3D1, cycle 3 day 1.

Early grade 3/4 neutropenia was defined as the occurrence of grade 3/4 neutropenia within the first 56 days of treatment (up to cycle 3 day 1) and was analyzed by logistic regression. Time to first grade 3/4 neutropenia was analyzed using Cox proportional-hazards regression; patients who did not develop grade 3/4 neutropenia were censored at their last treatment cycle. [a] Adjusted for baseline ANC (<3.6 vs $\geq 3.6\times10^3/mm^3$) and histological subtype (invasive carcinoma of no special type vs non-ductal). [b] *p*-value from the log-rank test. All associations shown are nominally significant ($p < 0.05$).

Table S5. Univariate associations between genetic variants and progression-free survival in patients with hormone receptor (HR)-positive and human epidermal growth factor receptor 2 (HER2)-negative locally advanced or metastatic breast cancer.

		Progression-free survival			
**SNP**	**n**	**Probability (95% CI) at 5-years**	**Unadjusted HR (95% CI)**	***p*-value (log-rank)**	**Bonferroni -adjusted *p*-value**
NR1I2 rs3814055				0.99	
CC	36	0.27 (0.05 - 0.49)	1		
CT	63	0.43 (0.29 - 0.57)	1.02 (0.59–1.77)		
TT	14	0.35 (0.06 - 0.64)	0.99 (0.43–2.26)		
NR1I2 rs2472677				0.28	
TT	39	0.36 (0.16 - 0.56)	1		
TC	56	0.38 (0.24 - 0.52)	1.02 (0 .59–1.75)		
CC	18	0.34 (NA)	0.54 (0.23–1.26)		
NR1I2 rs6785049				**0.003**	0.28
AA	33	0.28 (0.03 - 0.53)	1		
AG	49	0.21 (0.05 - 0.37)	1.66 (0.93–2.97)		
GG	31	0.67 (0.49 - 0.85)	0.53 (0.24–1.16)		
AA – AG vs GG[b]	82	0.24 (0.10 - 0.38)	2.59 (1.31-5.12)	**0.004**	0.38
NR1I2 rs3814058				0.63	
TT	58	0.33 (0.15 - 0.51)	1		
TC	45	0.34 (0.16 - 0.52)	1.18 (0.70–1.98)		
CC	10	0.59 (0.28 - 0.90)	0.75 (0.26–2.12)		
NR1I2 rs3732360				0.14	
TT	49	0.37 (0.17 - 0.57)	1		
TC	46	0.25 (0.07 - 0.43)	1.38 (0.81–2.34)		
CC	18	0.6 (0.36 - 0.84)	0.64 (0.28–1.49)		
NR1I2 rs1054190				0.20	
CC	88	0.3 (0.16 - 0.44)	1		
CT - TT	25	0.59 (0.39 - 0.79)	0.64 (0.33–1.26)		
NR1I2 rs7643645				0.39	
AA	48	0.36 (0.20 - 0.52)	1		
AG	51	0.33 (0.15 - 0.51)	0.88 (0.52–1.49)		
GG	14	0.34 (NA)	0.52 (0.20–1.35)		
SULT2A1 rs182420				0.95	
TT	63	0.33 (0.17 - 0.49)	1		
TC	44	0.38 (0.20 - 0.56)	1.08 (0.64–1.81)		
CC	6	0.5 (0.11 - 0.89)	0.94 (0.29–3.05)		
SULT2A1 s2637125				0.85	
GG	82	0.33 (0.19 - 0.47)	1		
GA - AA	31	0.47 (0.29 - 0.65)	1.06 (0.60–1.87)		
SULT2A1 CNV				0.82	
2 copies	80	0.33 (0.19 - 0.47)	1		

0-1 copies	33	0.47 (0.29 - 0.65)	0.94 (0.54–1.64)		
ABCG2 rs2231142				0.86	
GG	99	0.35 (0.21 - 0.49)	1		
GT	14	0.39 (0.12 - 0.66)	1.07 (0.51–2.25)		
CDKN1B s2066827				0.16	
TT	64	0.34 (0.18 - 0.50)	1		
TG	41	0.39 (0.19 - 0.59)	0.91 (0.53–1.56)		
GG	8	0.25 (NA)	2.12 (0.89–5.08)		
CYP3A4 s35599367				0.35	
GG	102	0.36 (0.22 - 0.50)	1		
GA	9	0.17 (NA)	1.46 (0.66–3.22)		
ABCB1 rs1045642				0.21	
GG	30	0.38 (0.13 - 0.63)	1		
GA	61	0.38 (0.20 - 0.56)	1.02 (0.55–1.88)		
AA	22	0.27 (0.07 -0.47)	1.68 (0.83–3.41)		
ABCB1 rs2032582				0.56	
CC	37	0.35 (0.08 - 0.62)	1		
CT - CA	60	0.36 (0.20 - 0.52)	1.15 (0.65–2.05)		
TT - TA	16	0.3 (0.06 - 0.54)	1.51 (0.71–3.19)		
ABCB1 rs1128503				0.84	
GG	36	0.37 (0.12 - 0.62)	1		
GA	57	0.33 (0.15 - 0.51)	1.19 (0.67–2.12)		
AA	20	0.39 (0.17 - 0.61)	1.14 (0.55–2.38)		
POR rs1057868				0.1	
CC	58	0.34 (0.18 - 0.50)	1		
CT	47	0.49 (0.33 - 0.65)	1.03 (0.60–1.77)		
TT	8	0.13 (NA)	2.36 (1.03–5.41)		
CC – CT vs TT[b]	105	0.37 (0.25 - 0.49)	0.43 (0.19-0.95)	**0.03**	1.00

SNP, single-nucleotide polymorphism; [b]recessive genetic model; Nominally statistically significant *p*-values are marked in bold.

Table S6. Univariate associations between genetic variants and overall survival in patients with hormone receptor (HR)-positive and human epidermal growth factor receptor 2 (HER2)-negative locally advanced or metastatic breast cancer.

		Overall survival			
**SNP**	**n**	**Probability (95% CI) at 5-years**	**Unadjusted HR (95% CI)**	***p*-value (log-rank)**	**Bonferroni -adjusted *p*-value**
NR1I2 rs3814055				0.78	
CC	36	0.47 (0.22 - 0.72)	1		
CT	63	0.44 (0.26 - 0.62)	1.28 (0.64–2.55)		
TT	14	0.38 (0.03 - 0.73)	1.13 (0.42–3.04)		
NR1I2 rs2472677				0.77	
TT	39	0.52 (0.34 - 0.70)	1		
TC	56	0.4 (0.20 - 0.60)	0.89 (0.47–1.71)		
CC	18	0.4 (0.05 - 0.75)	0.71 (0.28–1.82)		
NR1I2 rs6785049				**0.03**	1.00
AA	33	0.46 (0.19 - 0.73)	1		
AG	49	0.28 (0.08 -0.48)	1.45 (0.72–2.96)		
GG	31	0.66 (0.42 - 0.90)	0.49 (0.19–1.27)		
AA – AG vs GG[b]	82	0.34 (0.18 - 0.50)	2.61 (1.16-5.88)	**0.02**	1.00
NR1I2 rs3814058				0.29	
TT	58	0.54 (0.34 - 0.74)	1		
TC	45	0.32 (0.12 - 0.52)	1.65 (0.88–3.11)		
CC	10	0.5 (0.15 - 0.85)	1.19 (0.40–3.55)		
NR1I2 rs3732360				0.07	
TT	49	0.5 (0.28 - 0.72)	1		
TC	46	0.28 (0.08 -0.48)	1.75 (0.91–3.36)		
CC	18	0.67 (0.43 - 0.91)	0.70 (0.25–1.93)		
NR1I2 rs1054190				0.05	
CC	88	0.39 (0.23 - 0.55)	1		
CT - TT	25	0.56 (0.23 - 0.89)	0.41 (0.16–1.04)		
NR1I2 rs7643645				0.69	
AA	48	0.48 (0.30 - 0.66)	1		
AG	51	0.38 (0.16 -0.60)	0.76 (0.40–1.43)		
GG	14	0.56 (0.25 -0.87)	0.92 (0.34–2.44)		
SULT2A1 rs182420				0.69	
TT	63	0.41 (0.21 - 0.61)	1		
TC	44	0.47 (0.25 - 0.69)	0.97 (0.52–1.84)		
CC	6	0.39 (NA)	1.64 (0.49–5.49)		
SULT2A1 rs2637125				0.83	
GG	82	0.41 (0.25 - 0.57)	1		
GA - AA	31	0.50 (0.21 - 0.79)	0.92 (0.45–1.88)		
SULT2A1 CNV				0.96	
2 copies	80	0.41 (0.25 - 0.57)	1		
0-1 copies	33	0.51 (0.24 - 0.78)	1.03 (0.41–2.63)		

ABCG2 rs2231142				0.95
GG	99	0.42 (0.26 - 0.58)	1	
GT	14	0.55 (0.24 - 0.58)	0.97 (0.38-2.46)	
CDKN1B rs2066827				0.85
TT	64	0.47 (0.29 - 0.65)	1	
TG	41	0.37 (0.13 - 0.61)	1.04 (0.55–1.98)	
GG	8	0.44 (0.07 - 0.81)	1.37 (0.47–3.96)	
CYP3A4 rs35599367				0.35
GG	102	0.45 (0.31 - 0.59)	1	
GA	9	0.14 (NA)	1.56 (0.61–3.96)	
ABCB1 rs1045642				0.71
GG	30	0.36 (0.07 - 0.65)	1	
GA	61	0.47 (0.29 -0.65)	0.85 (0.42–1.72)	
AA	22	0.43 (0.18 -0.68)	1.17 (0.49–2.78)	
ABCB1 rs2032582				0.83
CC	37	0.42 (0.15 - 0.69)	1	
CT - CA	60	0.49 (0.31 -0.67)	0.81 (0.41–1.59)	
TT - TA	16	0.29 (NA)	0.91 (0.36–2.25)	
ABCB1 rs1128503				0.81
GG	36	0.44 (0.19 - 0.69)	1	
GA	57	0.42 (0.22 - 0.62)	0.90 (0.46–1.76)	
AA	20	0.49 (0.18 -0.80)	0.74 (0.30–1.84)	
POR rs1057868				0.41
CC	58	0.42 (0.24 - 0.60)	1	
CT	47	0.54 (0.36 - 0.72)	1.21 (0.63–2.32)	
TT	8	0.45 (0.08 -0.82)	2.02 (0.69–5.92)	

SNP, single-nucleotide polymorphism; [b]recessive genetic model. Nominally statistically significant *p*-values are marked in bold.

Table S7. Optimism-corrected performance of adjusted models assessing the association of *NR1I2* rs6785049 with severe neutropenia, progression-free survival, and overall survival.

**Model**	**Metric**	**Apparent**	**Mean optimism**	**Optimism corrected**
Grade 3/4 neutropenia	AUROC	0.747	0.018	0.729
Progression-free survival	Harrell C-statistic	0.653	0.022	0.631
Overall survival	Harrell C-statistic	0.697	0.022	0.675

AUROC, area under the receiver operating characteristic curve.

Table S8. Bootstrap 95% confidence intervals for associations of *NR1I2* rs6785049 with severe neutropenia, progression-free survival, and overall survival.

	**Overall survival**		**Progression-free survival**		**Grade 3/4 neutropenia**	
**Term**	**95% CI low**	**95% CI high**	**95% CI low**	**95% CI high**	**95% CI low**	**95% CI high**
NR1I2 rs6785049 (AA – AG versus GG)	1.28	8.15	1.48	6.75	1.42	14.12
Line of therapy	1.14	4.47	1.30	4.51		
Menopausal status	1.01	7.97				
Age at palbociclib initiation	0.97	4.22	0.99	3.41		
Histological subtype					0.08	1.07
Baseline ANC					0.04	0.43

ANC, absolute neutrophil count.

Table S9. Genotyping characteristics of the analyzed variants.

**Gene**	**rsID**	**Cat. #**		**[VIC/FAM]**	**Al·lel 1 (n, genotype)**	**Al·lel 2 (n, genotype)**	**Both (n, genotype)**	**MAF**	**HWE**
NR1I2	rs3814055	C__27504984_30	4362691	[C/T]	36, CC	14, TT	63, CT	0.40	0.09
	rs2472677	C__26079845_10	4351379	[C/T]	18, CC	39, TT	56, TC	0.41	0.77
	rs6785049	C__29280426_10	4351379	[A/G]	33, AA	31, GG	49, AG	0.49	0.16
	rs3814058	C__11231740_10	4351379	[C/T]	10, CC	58, TT	45, TC	0.29	0.76
	rs3732360	C__27472912_30	4351379	[C/T]	18, CC	49, TT	46, TC	0.36	0.20
	rs1054190	C___8760434_10	4351379	[C/T]	88, CC	1, TT	24, CT	0.12	0.65
	rs7643645	C___1834250_20	4351379	[A/G]	48, AA	14, GG	51, AG	0.35	0.94
SULT2A1	rs182420	C___8716099_10	4351379	[C/T]	6, CC	63, TT	44, TC	0.25	0.64
	rs2637125	C__26078613_10	4351379	[A/G]	2, AA	82, GG	29, GA	0.15	0.76
	CNV	Hs03013147_cn	4400291		80, 2copies	31, 1copy	2, 0copies		
ABCG2	rs2231142	C__15854163_70	4362691	[G/T]	99, GG	0, TT	14, GT	0.06	0.48
CDKN1B	rs2066827	C__11916245_10	4351379	[G/T]	8, GG	64, TT	41, TG	0.25	0.69
CYP3A4	rs2740574	C___1837671_50	4362691	[C/T]	1, CC	108, TT	4, TC	0.03	0.0008
	rs35599367	C__59013445_10	4351379	[G/A]	102, GG	0, AA	9, GA	0.04	0.66
ABCB1	rs1045642	C___7586657_20	4362691	[A/G]	22, AA	31, GG	61, GA	0.46	0.41
	rs2032582	C_11711720D_40	4362691	[C/T]	37, CC	16, CA-CT	60, AA-AT	0.41	0.29
	rs2032582	C_11711720C_30	4362691	[C/A]					
	rs1128503	C___7586662_10	4362691	[A/G]	20, AA	36, GG	57, GA	0.43	0.75
POR	rs1057868	C___8890131_30	4362691	[C/T]	58, CC	8, TT	47, CT	0.28	0.71

rsID, reference single-nucleotide polymorphism identifier; CNV, copy number variation; MAF, Minor Allele Frequency; HWE: Hardy–Weinberg Equilibrium

(a)

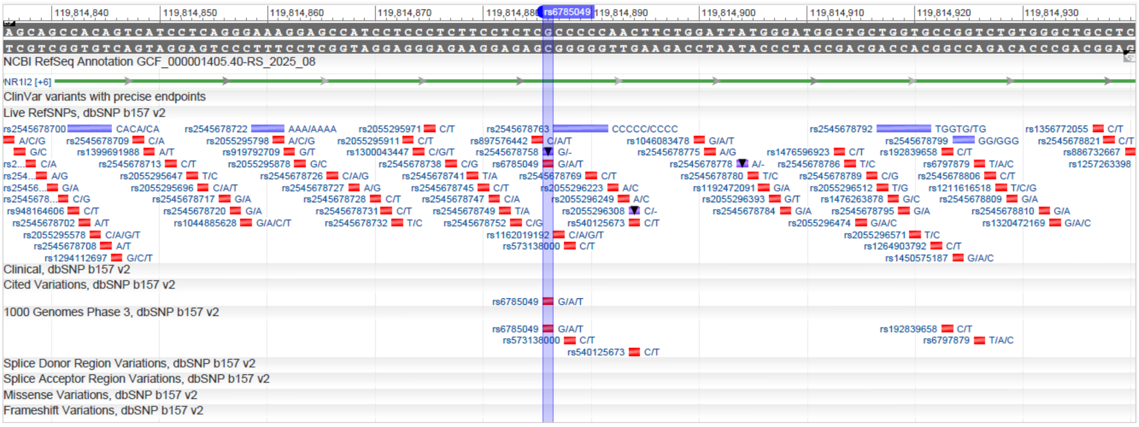

(b)

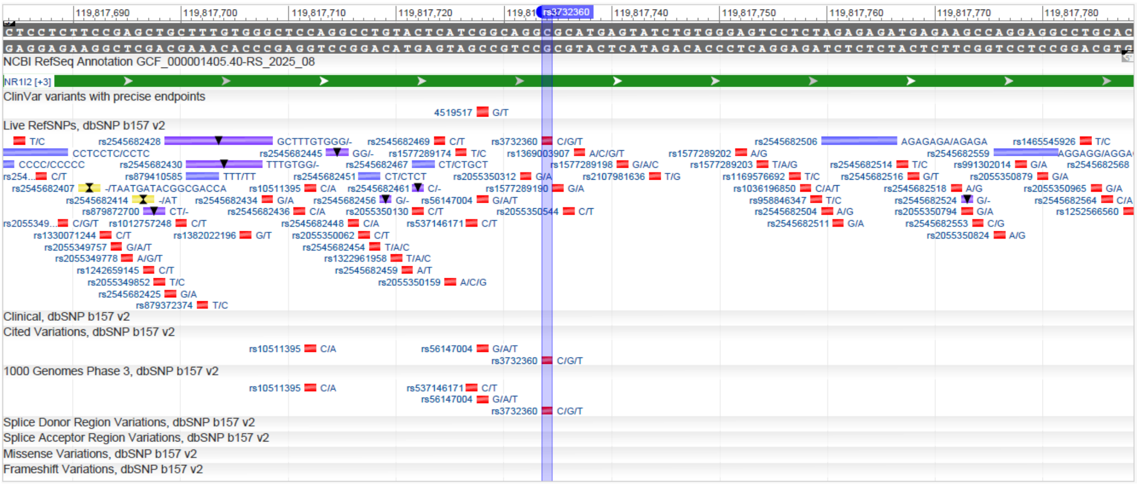

Figure S1. Genome browser views of NR1I2 rs6785049 and rs3732360. Genome browser screenshots showing the genomic location of rs6785049 (a) and rs3732360 (b), together with the corresponding genomic context and annotated genomic features. The views provide information on the position of each variant within the genomic region and its surrounding annotated elements. Data were obtained from the UCSC Genome Browser (http://genome.ucsc.edu).

(a)

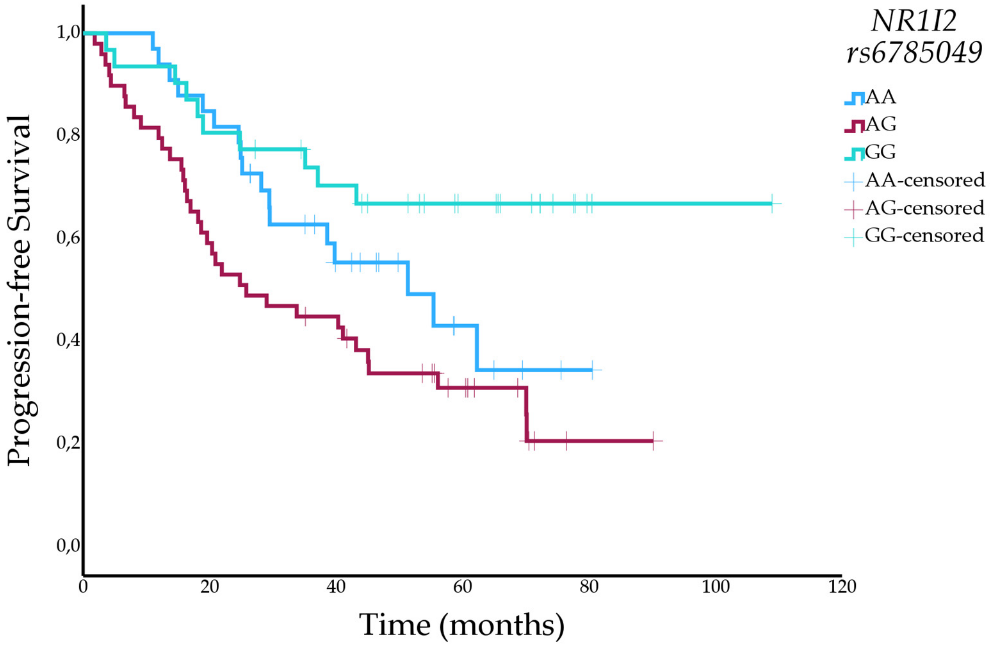

(b)

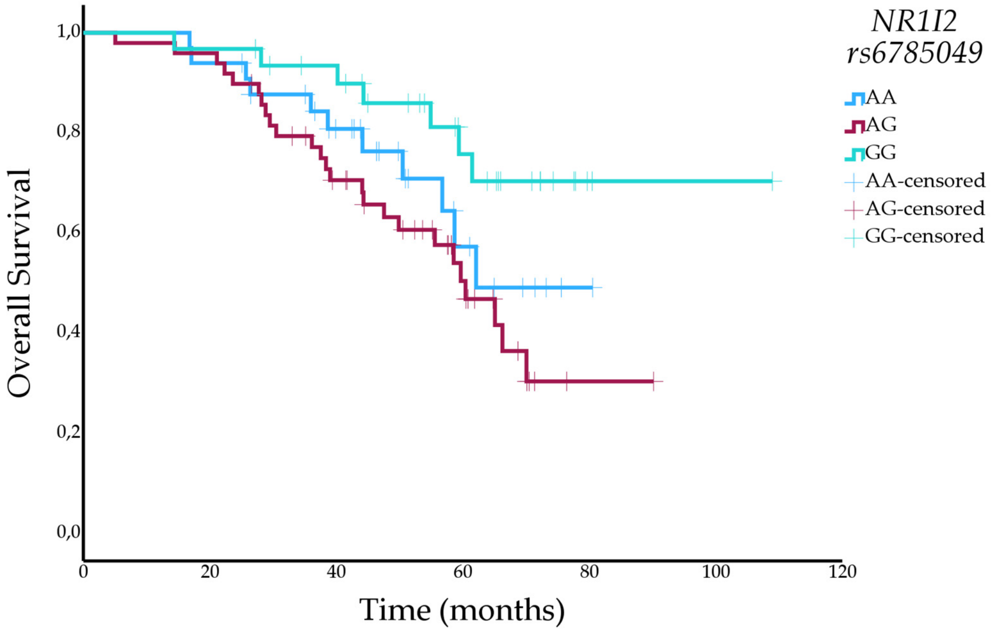

Figure S2. (a) Progression-free survival according to *NR1I2* rs6785049 genetic variant and (b) overall survival according to the *NR1I2* rs6785049 genetic variant in patients with hormone receptor (HR)-positive and human epidermal growth factor receptor 2 (HER2)-negative locally advanced or metastatic breast cancer.
